# Perspectives on Using *Physcomitrella* Patens as an Alternative Production Platform for Thapsigargin and Other Terpenoid Drug Candidates

**DOI:** 10.4137/pmc.s2220

**Published:** 2009-03-04

**Authors:** Henrik Toft Simonsen, Damian Paul Drew, Christina Lunde

**Affiliations:** VKR Research Centre Pro-Active Plants, Department of Plant Biology and Biotechnology, Faculty of Life Sciences, University of Copenhagen, Thorvaldsensvej 40, 1871 Frederiksberg C, Denmark

**Keywords:** physcomitrella, thapsigargin, drug production, sesquiterpene

## Abstract

To overcome the potential future demand for terpenoids used as drugs, a new production platform is currently being established in our laboratory. The moss *Physcomitrella* has been chosen as the candidate organism for production of drug candidates based on terpenoids derived from plants, with a primary focus on the sesquiterpene lactone, thapsigargin. This drug candidate and other candidates/drugs with sesquiterpene skeleton are difficult to obtain by chemical synthesis due to their large number of chiral centers. Furthermore, they are not available in sufficient amounts from their original plant. The requirement for a new production system to meet the potential market demand for these compounds is not only obvious, but also essential if sufficient quantities of the drug candidates are to be available for the potential therapeutic use.

## Introduction

Plant cells are capable of producing an overwhelming variety of secondary metabolites, both in terms of complexity and quantity. These small organic molecules allow plants to cope with various types of stress, and often have biological activities beneficial to humans that make them of high commercial interest to the biotechnology industry.^1^ Attempts have been made to make this overwhelming variety of compounds economically accessible by introducing the biosynthesis pathways of interesting natural products from higher plants into other organisms, for example yeast cultures.^2^ This perspective discusses the benefits of a recently established production organism, the moss *Physcomitrella patens*, as a host for the production of terpenoids.

Plants containing pharmaceutically active natural products have been used worldwide in folk medicine since at least 4000 BC.^3^ More than 80,000 of the 250,000 known species of higher plant are used in medicine,^4^ and since some of these plants are threatened by extinction, much effort has been put into identifying the active compounds before their natural source disappears. The plants have often been used without knowing the exact mechanism by which the active compound exerts its effect. This is still true for several well-known chemical groupings of natural products, such as certain saponins, flavonoids and ginkgolides from *Ginkgo biloba* and *Panax ginseng.*^5^ However, for an increasing number of plants the pharmacological activity has been linked to one or more specific metabolites. Plant used in traditional medicine has been subjected to many different studies, ranging from antimicrobial,^6^ antimalarial,^7^ and to more advanced studies including clinical trials of either extracts or isolated compounds.^8^ A range of these isolated natural products have been pursued as pharmaceutical drugs, and a limited number have been successful.^5^,^9^ Many of these biologically active compounds from plants are isoprenoid molecules. At the end of 2006, more than 50,000 isoprenoid structures had been identified, of which many have known biological activities.^10^ Several of these isoprenoids have anti-tumor activity, including the well known Taxol (paclitaxel), which is originally extracted from pacific yew trees.^9^ Novel drugs are often in high demand, which leads to a high harvest pressure on the source of the drug such as the wild plants. As has already been seen in the cases of Taxol and the anti-malaria drug, artemisinin, one cannot only rely on the wild population for the supply of drugs.^11^ There are several problematic issues regarding the collection of wild plants, and the fact that pharmaceutical companies need a secure and regular supply of plants if they are to be used for drug production, steers commercial research away from “difficult” plants, which cannot be cultivated with high yields.^12^ This is one of the major challenges for the development of the very promising drug candidate thapsigargin.^13^

A thapsigargin derived drug is currently undergoing clinical trials for the treatment of breast, kidney and prostate cancer.^14^,^15^ Thapsigargin induces apoptosis in mammalian cells by inhibiting the sarco/endoplasmatic calcium ATPase (SERCA), resulting in an elevated cytoplasmic Ca^2+^ level, which eventually leads to the death of the cell.^16^ The crystal structure of the SERCA pump in which thapsigargin was bound inside the transmembrane pore showed large conformational changes when compared with the structure of SERCA with bound Ca^2+^.^17^ This comparison provided some of the information needed for later design of the cell-targeted pro-drug based on thapsigargin that is now in clinical trials.^14^–^16^ At the present time all of the commercially available thapsigargin is obtained from intact fruits and roots of *Thapsia garganica* L. plants that are collected from the wild. The potential increasing worldwide demand for thapsigargin is endangering the sparse populations of *T. garganica* and other *Thapsia* species in the Mediterranean area and may lead to their extinction. *Thapsia* and Apiaceae plants in general are difficult to germinate from seed and to maintain under greenhouse conditions.^13^,^18^ Jäger et al.^19^ are the only researchers to have reported the establishment of *in vitro* cultures of *T. garganica* for the purpose of producing thapsigargins. They induced somatic embryos that accumulated two thapsigargins, namely nortrilobolid and trilobolid; the ability to convert somatic embryos into plantlets was not investigated. To date, there has been no report of the successful regeneration of *T. garganica* plantlets *in vitro.*^20^

Micropropagation of *T. garganica* has also been investigated as an option for conservation purposes as wild populations of the plants are becoming increasingly sparse.^13^ Results show that optimization of *in vitro* propagation of *Thapsia* for large scale production could be very difficult indeed. This is consistent with reports that acclimatization of plants from the Apiaceae family in general is very difficult to achieve.^18^,^21^ Nevertheless, *in vitro* propagation could serve as a way for the conservation of the medicinally important species, *T. garganica.*^20^ Another possible method by which thapsigargin could be obtained is by chemical synthesis. However, although thapsigargin can be chemically synthesized, it is not in any way commercially feasible at the moment.^22^

Given the difficulties associated with the supply of thapsigargin from wild plants, the inability to cultivate *Thapsia garganica* efficiently, and the expense of chemical synthesis, an alternative production platform needs to be developed to meet world market demand.^16^,^20^ Previously, attempts have been made to transfer the sesquiterpene biosynthetic pathway to bacteria or fungi; these have been somewhat successful.^10^ Recently the group of J. Keasling reported the successful generation of a yeast strain producing the artemisinin precursor amorphadiene.^2^ One of the major obstacles for metabolic engineering in microorganisms is to obtain functional expression of P450’s. Yeast is generally considered to be a better host for this, owing to its eukaryotic membrane structure.^23^ Simple pathways such as the three steps for the formation of amorphadiene are likely to be successful in yeast, whereas more complex pathways such as the 19 step taxol biosynthesis are much more complex and difficult to predict. With microbial engineering some of the critical elements for stable production are: Choice of host strain, expression level, codon-bias, N-terminal modifications and choice of reduction partners, all of equal importance.^23^,^24^

**Figure 1.**
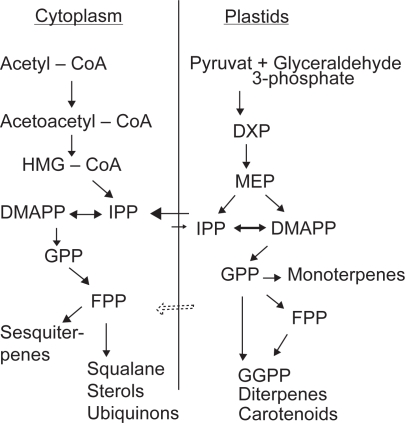
Overall sketch of the two IPP/DMAPP producing pathways and their intracellular localization in plants. Arrows indicate the overall pathway; many intermediates and additional substrates such as ATP and NADPH are omitted. The possible crosstalk between pathways is also marked with arrows. GPP: Geranyl diphosphate (C10), FPP: Farnesyl diphosphate (C15), GGPP: Geranylgeranyl diphosphate (C20).^3^,^51^

To achieve such success, an in-depth understanding of the original biosynthesis pathway of the target molecule is of great importance. The primary metabolism of terpenes and especially sesquiterpenes is gradually being elucidated and issues such as compartmentation (Fig. 1) have been observed to be of vital importance.^3^,^25^ When transferring biosynthetic pathways from one kingdom to another, the correct localization of introduced enzymes and other regulation issues can create problems as observed with plant P450s expressed in microorganisms.^10^,^23^ Although these problems can be overcome as they are encountered, it is possible that they could be avoided by using other host organisms like the moss, *Physcomitrella patens*, or by using cell cultures as is done with Taxol production.^12^ Attempts at using yeast strains for the production of amorphadiene, an artemisinin precursor have been somewhat successful, but for small natural products several obstacles have to be addressed as outlined above. Yeast utilizes several regulatory mechanisms to maintain tight control over the intracellular level of farnesyl diphosphate (FPP), the central precursor to nearly all yeast isoprenoid products and an essential building block for sesquiterpenes.^26^ High-level production of non-native terpenoids requires that FPP flux is diverted from production of sterols to the heterologous metabolic pathway. To do so, expression of the gene encoding squalene synthase (ERG9), the first committed step in sterol biosynthesis, can be down-regulated. Under certain culture conditions, the production of amorphadiene was increased five-fold by ERG9 repression.^26^ The isoprenoid pathway in the plastid and in the cytosol supplies precursor for mono- and sesquiterpene biosynthesis and an array of other compounds, many of which are crucial to plant growth and fit-ness such as sterols, gibberellins, carotenoids and chlorophyll.^27^ Thus, strong and constitutive expression of introduced genes could be most harmful. It is therefore highly recommended in future metabolic engineering experiments to direct gene expression to a specific tissue or organ or use an inducible system, as observed with taxadiene production in *Arabidobsis.*^28^

## The Benefits of the Moss *Physcomitrella Patens*

The moss lineage diverged from vascular plants about 450 million years ago and has maintained a small and rather simple vegetative body structure with few specialized cell types.^29^ Unlike vascular plants, the haploid gametophyte dominates the life cycle, while the diploid sporophyte is short-lived and completely dependent on the gametophyte.^30^ *Physcomitrella patens* has been used as a model for studies on plant evolution, development and physiology for more than 40 years. However, the recent release of the full genome sequence has sparked an enormous interest in this plant, which is also increasingly being used in biotechnology for safe production of complex biopharmaceuticals.^31^,^32^ One example is the production of the highly glycosylated peptide hormone erythropoietin (EPO).^33^ Production of EPO was carried out in a *Physcomitrella* Delta-fuc-t Delta-xyl-t strain, deficient in the enzymes responsible for the plant-specific core-bound alpha 1,3-fucose and beta 1,2-xylose. Thus, the final product will have a humanized glycosylation pattern and therefore be non-immunogenic.^33^,^34^

*Physcomitrella* has a number of features that make it an attractive production system when compared with other plant production hosts. *Physcomitrella* can be grown in sterile cultures using standard plant tissue culturing techniques. It grows photoautotrophically in a simple inorganic media without the need for phytohormones, vitamins or a carbon source.^35^ It can be maintained on solid media or as liquid cultures in flasks or fermenters. Unlike other plant production systems where liquid cultures are normally composed of cells from undifferentiated callus, large continuous *Physcomitrella* cultures can be maintained as protonema (the haploid phase).^36^ In contrast to callus cultures, in which undifferentiated cells have a high level of somaclonal variation, a culture consisting of protonema has a stable genetic background, ensuring that the genetic composition of continuous *Physcomitrella* cultures does not change over time.^31^ In addition, *Physcomitrella* has proved to be very tolerant to a number of abiotic stresses.^37^ This high level of tolerance could ensure that the *Physcomitrella* production strain will be less affected in fitness during the biosynthesis.

The protonemal state can be maintained by continuously splitting the tissue into smaller pieces using a propeller fitted with blades. Cultivation in suspension allows scaling up of biofermenters to several thousand liters and the minimal media, which contains only minerals and water, enables high volume and low cost, and minimizes the risk of contamination. An additional advantage is that unlike any other plant known to date, *Physcomitrella* performs homologous recombination with high efficiency.^38^ This allows efficient and precise gene deletions, replacements and insertions and permits us to make specific and controlled recombination events that generate transformants with predictable properties. Thus, development of a stable production strain does not require crossing steps or regeneration of whole plants.^39^ Combined with the Cre-lox technology, high throughput production strains with a defined background lacking the antibiotic resistance marker can be generated.^38^ The availability of several characterized constitutive or inducible promoters will further assist in the formation of useful production strains.^40^–^42^

Ease of handling, simple life cycle and growth conditions, and the unique molecular tools available for gene targeting make the moss *Physcomitrella* an ideal candidate for the production of natural products that are difficult to access through other means. A potential challenge in the production of high levels of secondary metabolites is secretion into the media. Wild type *Physcomitrella* is able to secrete large amounts of the tetracyclic diterpene, 16α-hydroxykaurane, which forms needle-like crystal structures when *Physcomitrella* grows on solid media in sealed containers.^43^ This demonstrates that the capacity exists within *Physcomitrella* for the processing of terpenoid metabolites through secretion of the compound in large quantities. To utilize this pathway one should think of a knock-out the original diterpene production.^44^

## Combinatorial Biosynthesis in Moss

Combinatorial biosynthesis has already been successfully employed in micro-organisms for the production of compounds such as novel antibiotics.^45^,^46^ An *in vivo* combinatorial biosynthesis approach is pursued in *Physcomitrella*, primarily to obtain new and known natural products with useful biological activity. The generation of a moss that produces thapsigargin would add to our library of mutants, and secure a stable supply of chemically related and robust drugs. Following the establishment of such a model of *Physcomitrella patens*, the production of thapsigargin could be further enhanced by utilizing the knowledge of carbon and other important atom fluxes within plant cells.

**Figure 2.**
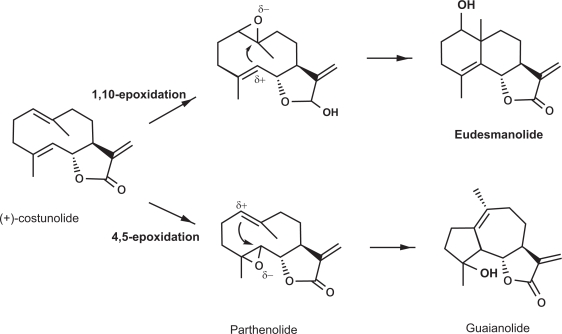
The proposed formation of eudesmanolides and guaianolides from costunolide in Chicory.^49^,^50^

In addition to thapsigargin and artemisinin, other sesquiterpene lactones are of interest as therapeutics. One such compound is parthenolide, a well-studied sesquiterpene lactone that is the major active compound in the medicinal plant *Tanacetum parthenium* (feverfew) and very promising as a cancer drug.^47^ *Tanacetum parthenium* is currently used as herbal remedy to relieve migraine, to help prevent blood clots, as an anti-inflammatory for relief in cases of arthritis, to relieve some types of menstrual problems, and as a digestive aid.^48^ Interestingly, parthenolide may be one of the precursors for thapsigargins (Fig. 2).^49^,^50^ Both artemisinin and thapsigargin are currently obtained by extraction from plants. Given their wide range of potential medical applications, the demand for these compounds is likely to vastly exceed the current levels of the current production. An important additional outcome of this concept is that knowledge obtained during the establishment of a *Physcomitrella* strain producing a thapsigargin precursor will enable the design of several other drug-candidate-producing moss strains, especially within the chemical class of sesquiterpene lactones.

With the results that we currently are verifying in our lab we believe that the first simple structures can be produced within one year, with increasing complexity of the molecules produced in the following years. In conclusion, the possibilities of using *Physcomitrella* as a new production platform for plant-derived natural products are numerous and interesting. While several issues need to be addressed and are currently being so, the future remains promising, and various kinds of natural products could be produced in this simple and easily grown plant.
